# Evolution of vertebrate interferon inducible transmembrane proteins

**DOI:** 10.1186/1471-2164-13-155

**Published:** 2012-04-26

**Authors:** Danielle Hickford, Stephen Frankenberg, Geoff Shaw, Marilyn B Renfree

**Affiliations:** 1 ARC Centre of Excellence for Kangaroo Genomics, Department of Zoology, The University of Melbourne, Victoria, 3010, Australia

**Keywords:** Marsupial, Tammar wallaby, Opossum, Gene cluster

## Abstract

**Background:**

Interferon inducible transmembrane proteins (IFITMs) have diverse roles, including the control of cell proliferation, promotion of homotypic cell adhesion, protection against viral infection, promotion of bone matrix maturation and mineralisation, and mediating germ cell development. Most IFITMs have been well characterised in human and mouse but little published data exists for other animals. This study characterised IFITMs in two distantly related marsupial species, the Australian tammar wallaby and the South American grey short-tailed opossum, and analysed the phylogeny of the IFITM family in vertebrates.

**Results:**

Five IFITM paralogues were identified in both the tammar and opossum. As in eutherians, most marsupial *IFITM* genes exist within a cluster, contain two exons and encode proteins with two transmembrane domains. Only two *IFITM* genes, *IFITM5* and *IFITM10*, have orthologues in both marsupials and eutherians. *IFITM5* arose in bony fish and *IFITM10* in tetrapods. The bone-specific expression of *IFITM5* appears to be restricted to therian mammals, suggesting that its specialised role in bone production is a recent adaptation specific to mammals. IFITM10 is the most highly conserved IFITM, sharing at least 85% amino acid identity between birds, reptiles and mammals and suggesting an important role for this presently uncharacterised protein.

**Conclusions:**

Like eutherians, marsupials also have multiple IFITM genes that exist in a gene cluster. The differing expression patterns for many of the paralogues, together with poor sequence conservation between species, suggests that *IFITM* genes have acquired many different roles during vertebrate evolution.

## Background

**Figure 1:**
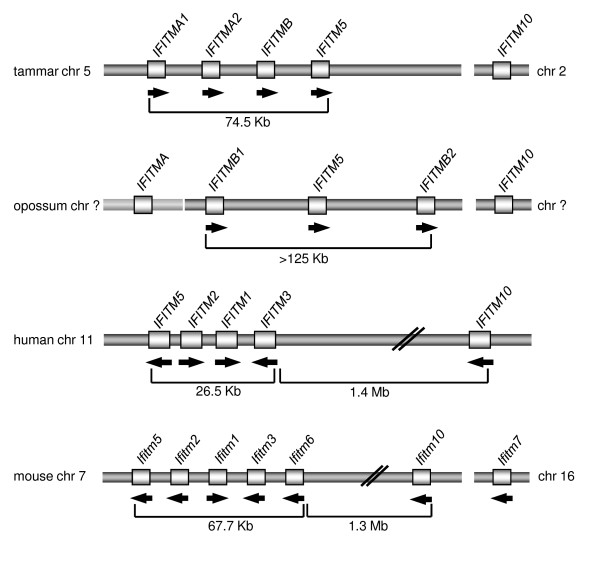
**Arrangement of IFITM gene clusters in the tammar, opossum, human and mouse.** Arrows indicate the direction of transcription. The paler bar for *IFITMA* in the opossum indicates that there is not enough information available to determine whether the contig containing *IFITMA* is continuous with the cluster containing *IFITMB1*, *-B2* and −*5.*

The human *ntereronnducibleransembrane* (*IFITM*) genes were originally identified by their differential response to stimulation by interferon [1,2], mediated by interferon-stimulated response elements (ISREs). The family consists of five genes (*IFITM1* (= *Leu-13* or *927*), *IFITM2 (1-8D)IFITM3 (1-8U)IFITM5* and *IFITM10*) all on chromosome 11 (Figure 1). All five genes encode proteins with 125–133 amino acids and two transmembrane domains and *IFITM1*, -*2* and −*3* have ISREs just 5' to their start codons [3]. Numerous biological roles have been attributed to IFITM1, -2 and −3 but little is known of the roles of IFITM5 and IFITM10 in humans. IFITM1 is expressed by leukocytes and endothelial cells, has anti-proliferative effects and promotes homotypic cell adhesion [4-6]. IFITM3 also inhibits cell proliferation [7], while IFITM2 induces both cell cycle arrest and subsequent p53-independent apoptosis in numerous cell lines [8]. IFITM proteins are also important components of the interferon-mediated innate immune system, and interest in these proteins has increased recently with the discovery that IFITM1, -2 and −3 offer protection against numerous viruses, including Influenza A, Dengue and HIV [9-11]. They may also act as tumor suppressors because of their ability to control the cell cycle. Supporting this idea, cells transitioning from a normal to a pre-malignant state often exhibit abnormal *IFITM* expression [12].

The *Ifitm* gene family has been well characterised in mice. *Ifitm3* (also known as *Mil1* and *Fragilis*), the first murine *Ifitm* gene to be characterised, was identified by a screen for genes expressed specifically in early primordial germ cells (PGCs) [13] and also in a subtractive cDNA screen comparing migrating PGCs with inner cell mass cells [14]. *Ifitm3* is on mouse chromosome 7, together with *Ifitm1* (*Mil2, Fragilis 2*), *Ifitm2* (*Mil3, Fragilis 3*), *Ifitm5* (*Fragilis 4*), *Ifitm6* (*Fragilis 5*) [15] and *Ifitm10* (also known as *6330512M04Rik*), whereas *Ifitm7* (*Mil4*) is on mouse chromosome 16 [14]. Murine *Ifitm* genes encode proteins with 104–144 amino acids and two transmembrane domains. *Ifitm1-3* and −*6* also have ISREs [14,15]. Mouse *Ifitm1-2* and −*3* genes are not respectively orthologous to human *IFITM1-2* and −*3* genes, and sequence comparisons between human and mouse *Ifitm* genes suggest that duplication of the *Ifitm* genes occurred independently in human and mouse lineages [15].

*Ifitm5* was renamed *bone restricted Ifitm-like* protein (*Bril*) because its expression in both humans and mice is restricted to bone, especially osteoblasts, and functional studies in mice suggest a role in bone matrix mineralisation and maturation [16]. Nothing is known of the functions of *Ifitm6-7* or −*10*. During murine embryonic development, *Ifitm1* is expressed in the extraembryonic and embryonic mesoderm until the mesoderm differentiates [14,15]. *Ifitm2* is ubiquitously expressed from E8.5, and *Ifitm3* expression mirrors the development of the germ cell lineage: it is expressed in the proximal epiblast from E5.5 and its expression is gradually restricted to PGCs as gastrulation proceeds [13-15]. *Ifitm5* and −*6* are not expressed in the embryo between E5.5 and E9.5 [15] but *Ifitm5* is expressed from E14.5 in developing bone [16].

*Ifitm3* expression delineates cells competent to become PGCs as early as E6.25 and it may have a role in germ cell development, possibly by promoting the formation of a discrete cell population that distinguishes presumptive PGCs from somatic cells via homotypic cell adhesion [13]. *Ifitm1* and −*3* may also control PGC migration. Application of ectopic *Ifitm1* and −*3* or silencing of *Ifitm1* via short hairpin RNA (shRNA) knockdown in embryos results in abnormal PGC migration [17]. *Ifitm1* could also be required for somite epithelialisation and formation of paraxial mesoderm, as these processes are defective when *Ifitm1* is silenced in vivo by RNA interference [18]. Knockdown of *Ifitm5* in osteoblast cell lines in vitro results in reduced bone mineralisation, implicating *Ifitm5* in matrix mineralisation and maturation [16]. Mice homozygous null for *Ifitm5* have smaller skeletons than heterozygous or wild type mice but their relatively mild phenotype suggests that the function of *Ifitm5* can be compensated for by some other factor [19]. The possibility of redundancy of *Ifitm* genes is supported by another study in which the locus containing the *Ifitm* cluster (*Ifitm1-2-3-5* and −*6*) was deleted by floxing out a 120-kb region. The resulting mice, even those homozygous for the deletion, apparently developed normally and were fully fertile, suggesting that these *Ifitm* genes are not essential for normal germline development or any other developmental process [20] and that their roles can be compensated for by other genes.

The studies described above focused on human or mouse IFITMs, and aside from a rudimentary description of *IFITM* genes in the cow and rat [15], the only other published descriptions of *IFITM* genes are for the electric eel [21] and trout [22]. Interest in IFITMs in humans has increased with the discovery of their roles as anti-viral agents and as markers of cancer and inflammatory diseases [12]. Such roles have not been described in the mouse, in which the main focus on Ifitms has been with respect to their role(s) in the development of the germ cell lineage. The lack of sequence conservation of IFITMs between human and mouse suggests that their roles may vary between species. Marsupials diverged from eutherians approximately 160 million years ago [23] and the Australian and South American marsupials have evolved independently since the break-up of Gondwana approximately 80 million years ago [24]. Comparing marsupial and eutherian *IFITM* genes will provide new information on the conservation and evolution of this gene family in mammals. This study therefore describes *IFITM* genes in two marsupial species, the Australian tammar wallaby *Macropus eugenii* (a macropodid marsupial) and the South American short-tailed grey opossum *Monodelphis domestica* (a didelphid marsupial) and compares them with existing genomic databases.

## Results

### Marsupial *IFITMs*

The tammar and opossum genomes each have five *IFITM* paralogues (Figure 1). Tammar *IFITM* genes identified were *IFITMA1* (Genbank accession number JQ254908), *IFITMA2* (JQ254909), *IFITMB* (JQ254910), *IFITM5* (JQ254911) and *IFITM10* (JQ254912), encoding predicted protein sizes of 12.1 to 16.8 kDa. Opossum *IFITMs* identified were *IFITMA, IFITMB1,IFITMB2* (Additional file 1: Table S 1), *IFITM5* (XM_001363778, previously identified as interferon-induced transmembrane protein 5-like) and *IFITM10* (XM_001367690, previously identified by Genbank as CD225 family protein FLJ76511-like), encoding predicted protein sizes ranging from 12.3 to 14.5 kDa. The only evidence of an opossum orthologue to tammar *IFITMA* is a short (796 nt) sequence (ti:515911565) in the opossum trace archives that contains both coding exons. There are several gaps in the current opossum genome assembly (Broad/monDom5) within the *IFITM* locus, so *IFITMA* may well be located within the *IFITM* cluster.

**Figure 2:**
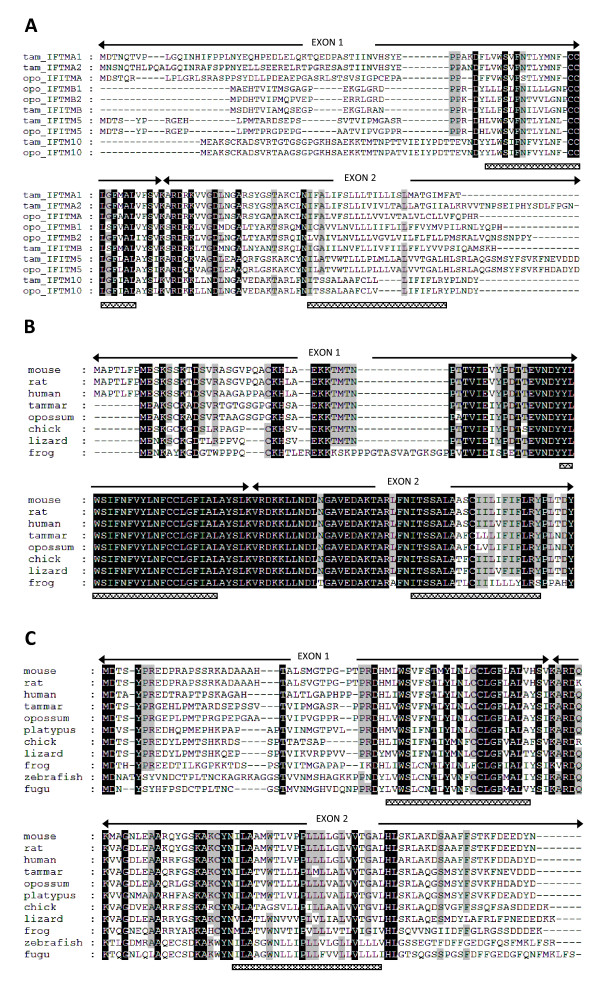
**IFITM protein alignments. A**, alignment of marsupial *IFITM* paralogues. **B**, **C**, alignments of *IFITM10* and *IFITM5* orthologues, respectively. Black shading represents identical amino acids conserved between all species, grey amino acids conserved in at least 75% of the species. Hatched bars underneath each alignment indicate the location of transmembrane domains.

Marsupial *IFITMs* contain two exons and encode two transmembrane domains. The highest level of sequence conservation occurs within the first transmembrane domain and in between the two transmembrane domains (Figure 2A), similar to eutherian *IFITM* genes*.* Conservation of IFITM protein sequences both between and within the two marsupial species is quite low (22-38% similarity) with the exception of the comparisons listed in Table 1. The motif AGGAAATAGAAACT is an interferon stimulated response element (ISRE) in human *IFITM1*[2] and tammar *IFITMA1* and *IFITMA2* both have an identical putative ISRE (AGGAAATAGAAAGT) located close (299 and 193 nucleotides respectively) to the start of their open reading frames. No such motifs were identified in the other tammar *IFITMs* or in any of the opossum *IFITMs*, although minimal 5′ sequence is available for opossum *IFITMA*.

**Table 1:** Pairwise comparison of the most highly conserved marsupial IFITM proteins and summary of the length of these proteins

**Genes and species**	**% AA similarity**
Tammar IFITMA1 (130 AA) - tammar IFITMA2 (152 AA)	73
Tammar IFITMA1 (130 AA) - opossum IFITMA (129 AA)	55
Tammar IFITMA2 (152 AA) - opossum IFITMA (129 AA)	55
Opossum IFITMB1 (110 AA) - opossum IFITMB2 (115 AA)	64
Tammar IFITMB (109 AA) - opossum IFITMB1 (110 AA)	68
Tammar IFITMB (109 AA) - opossum IFITMB2 (115 AA)	60
Tammar IFITM10 (123 AA) - opossum IFITM10 (123 AA)	96
Tammar IFITM5 (133 AA) - opossum IFITM5 (133 AA)	87

AA, amino acids.

**Figure 3:**
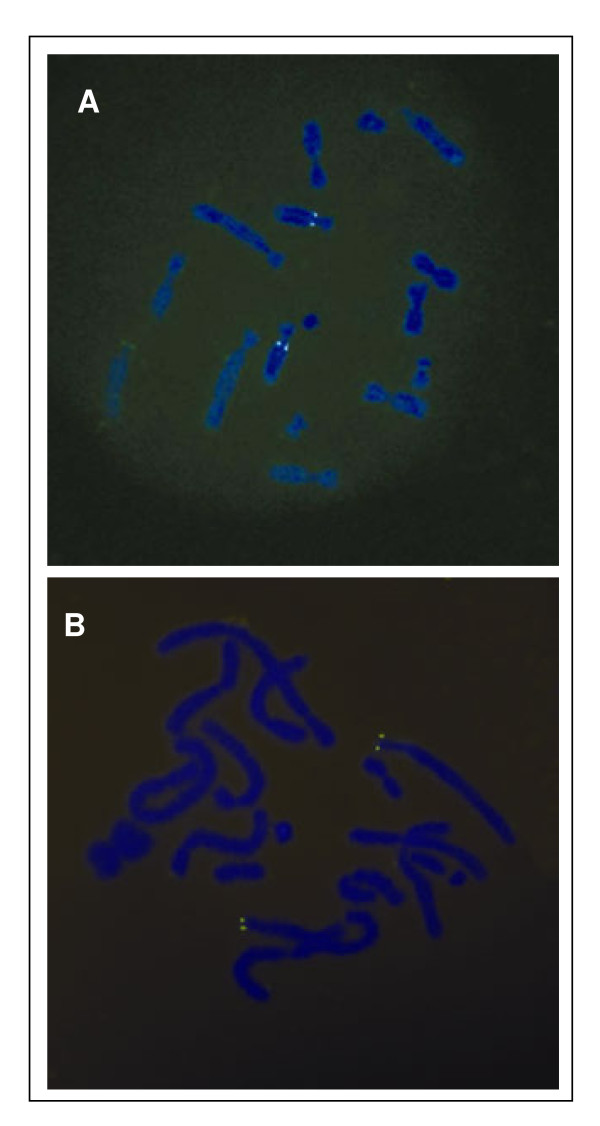
**FISH (fluorescence in situ hybridisation) for tammar *IFITMs. *A**, the *IFITM* gene cluster is located on chromosome 5q. **B**, the remaining single *IFITM* (*IFITM10*) is on chromosome 2p.

Screening of the tammar BAC library with probes against *IFITMA2* and *IFITM10* yielded two positive clones. Sequencing of the *IFITMA2*-positive BAC clone showed that *IFITMA1*, *-A2*, *-B* and −*5* are clustered together within a 74.5 kb region and share the same orientation (Figure 1). Using fluorescence in situ hybridisation (FISH), the *IFITM* cluster was localised to chromosome 5q and *IFITM10* to chromosome 2p (Figure 3). The chromosomal location of the opossum *IFITM* genes is unknown but at least *IFITMB1*, *-B2* and −*5* are clustered, whereas *IFITM10* is not within the main *IFITM* gene cluster. The location of *IFITMA* relative to the *IFITM* cluster is unknown.

Many of the human and some of the mouse *IFITM* genes have multiple transcripts, which differ either in their 5′, 3′ or both 5′ and 3′ untranslated regions (UTRs). Northern blots were attempted for each tammar *IFITM* gene but were unsuccessful (data not shown); probes to *IFITMA1* and *-A2* cross-reacted with each other whereas no distinct bands were obtained for *IFITMB-5* or −*10*, despite the use of multiple tissue samples and different probes. Therefore each tammar *IFITM* gene was used to search the tammar transcriptome and EST (expressed sequence tag) databases [25]. No transcripts representing *IFITM5* or *IFITM10* were identified. Two sequences containing at least the second exon and the polyA signal of *IFITMA2* and one for *IFITMB* were obtained and all supported the 3′ UTR identified by the BAC sequencing. There were four matches for *IFITMA1*, with two supporting a short 3′ UTR and the other two suggesting longer 3′ UTRs, only one of which contained a polyA signal. Thus, *IFITMA1* appears to have several transcripts. A similar analysis for *IFITMA2* also suggests several transcripts – whether these differ in the length of their 5′ or 3′ UTRs or contain additional exons upstream of the ones presented here is not known. The human and mouse genomes also each contain several *IFITM* processed pseudogenes. Searching the tammar genomic database [25] using tammar *IFITM* genes yielded one match for each gene, and in each instance this sequence contained an intervening intron.

**Figure 4:**
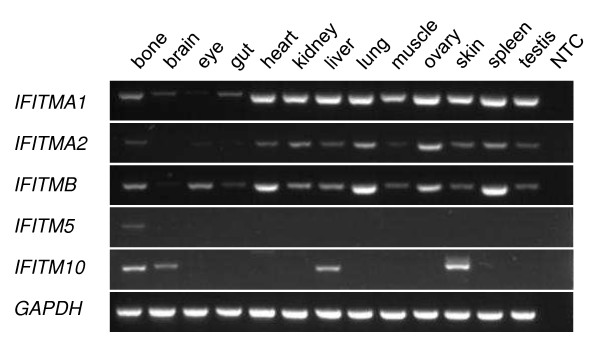
**RT-PCR of *IFITM* genes in tammar adult organs.** NTC, no template control.

**Figure 5:**
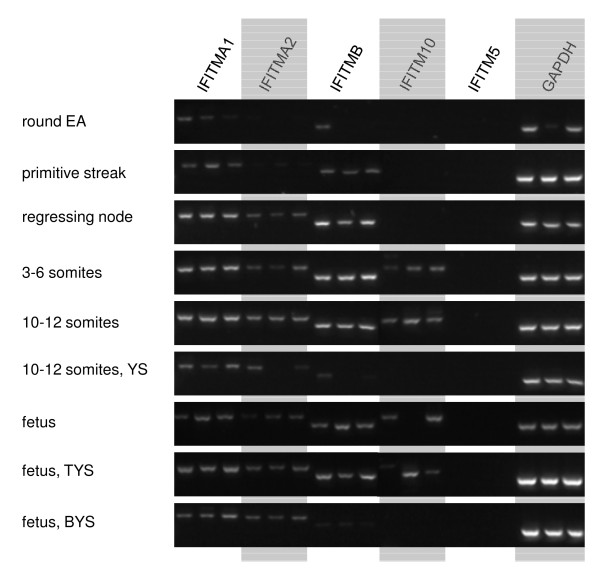
**RT-PCR of *IFITM* genes in tammar embryos and fetuses.** EA, embryonic area (epiblast); YS, yolk sac; TYS, trilaminar (vascular) yolk sac; BYS, bilaminar (avascular) yolk sac.

The expression of *IFITM* genes in the tammar was examined in adult tissues and also in peri-gastrulation and fetal stages by RT-PCR. In adult organs, *IFITMA1*, *-A2* and *-B* were widely expressed (Figure 4), whereas *IFITM5* is only expressed in bone. *IFITMA1*, *-A2* and *-B* were also the most widely expressed (temporally) *IFITM* genes during embryonic and fetal development (Figure 5), although *IFITMB* expression was very low in bilaminar (avascular) yolk sac (BYS). *IFITM10* expression was also absent from the BYS and was not detectable until slightly later in development, during early somitogenesis. *IFITM5* was not expressed at any pre-natal stage examined.

### IFITMs in other taxa

**Figure 6:**
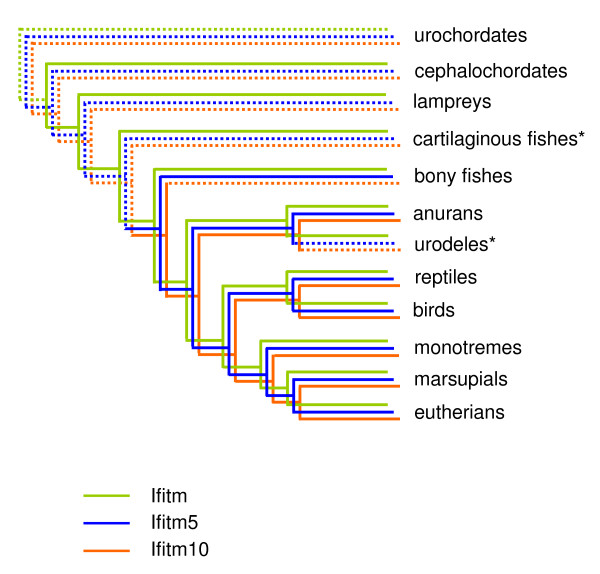
**Model for the evolution of *IFITM* genes in chordates.** The *IFITM* gene (green line) in the lamprey is only a partial transcript. In some clades such as anurans, bony fish, marsupials and eutherians, there are several *IFITM* paralogues, presumably arising through gene duplication. Bony fish are the first class in which *IFITM5* (blue line) is present, whereas *IFITM10* (red line) is tetrapod-specific. Note that in the cartilaginous fishes and urodeles (for which only EST data is available), *IFITM* orthologues listed as not present (dotted lines) may in fact exist but have not been detected due to the lack of a comprehensive genomic database. Extensive searching of the platypus (a monotreme) genome yielded a full transcript for *IFITM5*, but only partial transcripts for *IFITM* and *IFITM10*. *this information is based only on EST data because a genomic database is not available.

*IFITM* genes were identified in representatives from all seven major vertebrate classes (Figure 6, Table 2). There is no genome database available for urodelian amphibians and only a rudimentary one for chondrichthyes (cartilaginous fishes) so results in Figure 6 and Additional file 1: Table S 1 are based on EST data. An IFITM protein has also been described in the cartilaginous electric ray *Torpdeo marmorata*[21]. Only one small IFITM protein of approximately 30 amino acids was found for the lamprey (agnathan) and this aligns to *IFITM1* and −*3*-like *IFITM* genes from the turkey, zebrafish and frog with a higher score than it does to any *IFITM5* or −*10* orthologue (data not shown). *IFITM5* appears to have arisen in bony fish, and *IFITM10* in tetrapods. The apparent lack of either of these genes in urodeles probably reflects the paucity of genomic data currently available.

**Table 2:** Number, size and similarity of IFITM paralogues in different vertebrates

**Species**	**Number of paralogues**	**AA length**	**% AA ID between paralogues**
Mouse	6	104-144	23-73
Rat	6	104-144	49-84
Human	5	125-133	29-86
Tammar	5	109-152	23-73
Opossum	5	110-133	23-64
Platypus	3*	132	-
Frog	6	122-151	22-62
Fugu	4	121-138	21-95
Chicken	3	113-131	25-32
Lizard	3^	120-135	23-35
Lamprey	1^#^	-	-

* full sequence is available for one IFITM paralog but only partial sequence is available for two other IFITM paralogues.

^ only partial sequence available for one paralogue.

# only partial sequence for a single paralogue available.

AA, amino acids; ID, identity.

**Figure 7:**
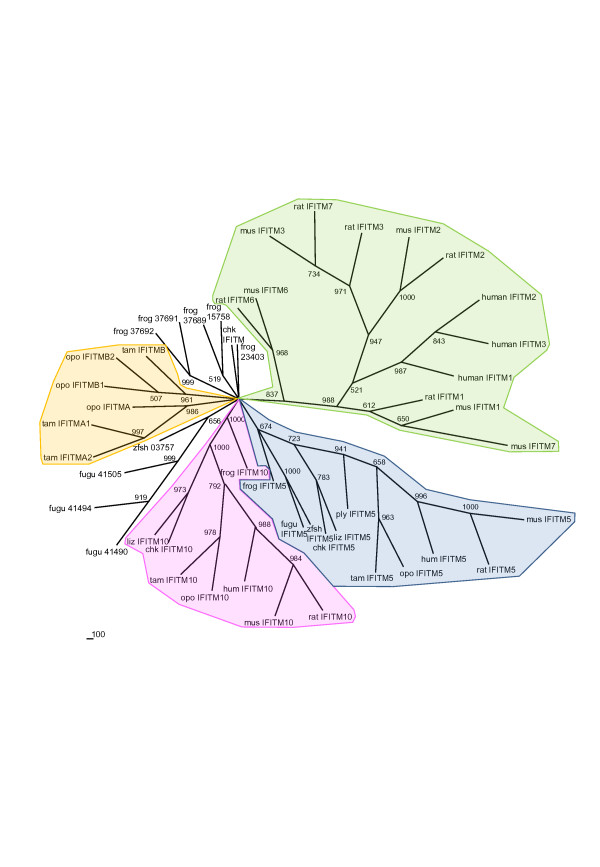
**Phylogenetic tree for vertebrate IFITM proteins.** The tree was constructed using the Neighbourhood joining method with 1000 bootstraps. Green shading denotes the cluster of general eutherian IFITMs, orange the marsupial IFITM cluster, pink the IFITM10 cluster and blue the IFITM5 cluster. Only IFITMs for which complete (or almost complete) protein sequence was available were included in this analysis. Opo, opossum; tam, tammar; mus, mouse; chk, chicken; zfsh, zebrafish; liz, lizard; ply, platypus.

A Neighbour joining phylogenetic tree constructed using IFITM amino acid sequences showed four distinct IFITM clusters (Figure 7). Two of these contained orthologues of IFITM5 and IFITM10 respectively from many different species, the third and fourth clusters contained the remaining eutherian and marsupial IFITMs respectively. The IFITM10 orthologues are the most highly conserved, exhibiting > 61% amino acid similarity between all species (increasing to > 85% similarity if frog IFITM10 is excluded). The IFITM5 orthologues share > 32% amino acid similarity (> 54% if fish and frog IFITM5s are excluded).

## Discussion

This study is the first to describe *IFITM* genes in marsupials. Marsupial *IFITM* genes are similar to those in eutherians with respect to their size (approximately 12 to 17 kDa), structure (two exons that encode two transmembrane domains) and their arrangement within a gene cluster. It also appears that like in mouse and human, there may be multiple transcripts for some of the tammar *IFITM* genes. Two tammar *IFITM* genes have a potential ISRE, similar to eutherian *IFITMs*. The apparent lack of any ISREs in opossum *IFITMs* is probably an artifact of lack of sufficient 5′ sequence for *IFITMA*, rather than an actual absence of this motif. ISREs may not be essential for *IFITM* expression; murine *Ifitm3* has two ISREs [15] but mutation of both motifs does not affect the expression of *Ifitm3* in the gastrula stage mouse embryo or in fetal gonads [17]. In the tammar several *IFITMs* are expressed during gastrulation, raising the possibility of a role for *IFITM* genes in marsupial germ cell development, although localisation of the transcripts is required before any conclusions can be drawn.

Although *IFITM* genes have been annotated in the genomes of numerous mammalian species (reviewed by [12]), IFITMs from only three mammalian species, the human, mouse and rat, were used for the current comparison. The mouse and human were chosen both because their IFITMs have been well studied and also because these species diverged from each other about 80 million years ago [26], approximately the same length of time as the split between Australasian and South American marsupials [24]. The rat was included to allow comparison of *IFITM* genes between two closely related species.

Phylogenetic analysis of IFITMs in various vertebrates has been undertaken previously [12] but the current analysis has expanded the data, including all identified marsupial IFITMs and also amphibian IFITMs. In contrast to [12], we also included IFITM10 paralogues. The definition of an IFITM within the literature is vague. All IFITMs contain the human leukocyte antigen cluster of differentiation CD225, yet not all proteins that contain CD225 are IFITMs. The sequences classified as IFITMs by [12] for their analysis included CD225-containing genes that had 2 exons and encoded proteins with a transmembrane domain. These proteins range from 102–157 amino acids in length, with a median length of 132 amino acids. Marsupial, chicken, lizard and rat *IFITM10* orthologues all consist of 2 exons and encode proteins 120–130 amino acids long. Mice and humans each have several *IFITM10* transcripts, which vary in the number of exons they contain, similar to other IFITM paralogues in these species. Thus, we decided to include IFITM10 paralogues as bona fide IFITM family members.

Our phylogenetic analysis showed that only *IFITM5* and *IFITM10* have clear orthologues in a range of vertebrate taxa. The orthologous relationships among other *IFITM* genes are often not very clear. A previous phylogenetic analysis of *IFITM* genes concluded that there is a high probability of primate-specific gene duplications [12], although concerted evolution cannot be ruled out as an explanation for the higher sequence similarity between paralogues rather than orthologues for many of the IFITMs. The low conservation among *IFITM* genes, even between species from the same subclass, suggests that either the roles of the different *IFITM* genes are not conserved between species or, more likely, that there is redundancy between them. The latter idea is strongly supported by an experiment in which the entire *IFITM* locus was deleted in mice without any apparent effects on normal development [20]. In fact, the study by [20] also suggests that at least during embryonic development, the IFITM family itself may be redundant. Further work is needed to clarify how critical IFITMs are for both embryonic development and general survival. The rapid evolution of IFITMs could be linked to their capacity to act as anti-viral agents; frequent mutation and duplications of these genes may act to counteract virus adaptations [12].

The lack of sequence conservation is mirrored by the lack of conservation of the expression patterns of the different *IFITM* genes between tammar, mouse and human. Expression data for human and mouse *IFITM* genes is available from the Unigene expressed sequence tag collection database [27] and some RT-PCR data is also available for the mouse [20]. Even the expression pattern of the most highly conserved *IFITM*, the tetrapod-specific *IFITM10*, differs between the three species although expression is more similar between human and tammar than either of these species are to mouse. In mouse *Ifitm10* is expressed in the brain and spleen, yet it is absent from these tissues in both the tammar (Figure 4) and human. Conversely, *IFITM10* is expressed in bone in tammar and human but not in mouse. The exceptionally high sequence conservation suggests that this gene has an important and conserved function, making it hard to reconcile the apparent lack of conservation in expression patterns. It is also curious that there is almost no information available on this gene in any species: the exception is a brief mention that *Ifitm10* ESTs are over-represented in the mouse brain [28].

Examining the expression of *IFITM5* in various taxa suggests that both the bone-specific expression of *IFITM5* [16, and this study] and the specialised role of *IFITM5* in bone production [16] is a recent adaptation specific to therian mammals. It would be interesting to examine *IFITM5* expression in tissues from a monotreme mammal but such samples were not available for this study. In zebrafish, in contrast to mammals, *IFITM5* is absent from bone, and instead is present in the brain, muscle and liver. The chicken EST database [27] does not include bone but does show high *IFITM5* expression in liver, muscle and spleen, with lower levels in the ovary and brain. The expression patterns of *IFITM5* in reptiles and amphibians are unknown.

## Conclusions

In conclusion, this study has described *IFITM* gene clusters in two marsupial species and has found evidence to demonstrate that the bone-specific expression of *IFITM5* is specific to mammals. It is also the first to recognize the exceptionally high sequence conservation of *IFITM10* between different taxa, which suggests an important and conserved (but as yet unidentified) role for *IFITM10*. This study also suggests that *IFITM* genes have acquired many different roles during their evolution in vertebrates.

## Methods

### Bioinformatics

*IFITMs* in the opossum genome were identified using the BLAT search engine on the UCSC Genome Bioinformatics website [29]. The region including and surrounding opossum *IFITM5* (chrUn:15587203–16132202, Oct. 2006 (Broad/monDom5) incorporating ~545 kb of sequence) was searched for predicted genes using GENSCAN [30]. Paralogues of *IFITM5* were identified by using Ensembl [31]. Primers based on human or opossum *IFITMs* were used to amplify tammar *IFITMs* by RT-PCR. The resulting products were cloned and sequenced and these sequences were then used to search the *M. eugenii* whole genomic shotgun (WGS) trace archives using BLASTn and tBLASTn [32] with discontiguous megablasts. This usually yielded small contigs containing either the first or second exon of each gene. These contigs were assembled using the online CAP3 program [33]. Sequence from these was then used to design tammar-specific primers within the 5 and 3 prime UTRs of each gene (Table 3). The ORF of each tammar *IFITM* gene was amplified by RT-PCR, cloned and sequenced.

**Table 3:** **Primers and RT-PCR conditions used to amplify tammar* IFITMs***

Gene	Forward	Reverse	Number of PCR cycles	Annealing temperature
*IFITMA1*	GTGCTCTGCGCTCCCTGT	AGCCATTCCCCAACAAATA	35	58*
*IFITMA2*	GGCTCGCAGTGCTACAGTCT	AAGTGAAGAGAGGCCACACC	30	60*
*IFITMB*	AGGAGCGCTATCCTGTGTGT	CTAGGGAAGCATGCAGGTGT	35	59*
*IFITM10*	CCTTCAGCAGCCTCCTCAG	AGTTCAGGTCAGTGGGATGG	40	58*
*IFITM5*	GACCTATGCGGCGAGTCCGC	GAAAGGAAGGAGGGCATCTT	40	58*
*GAPDH*	CCTACTCCCAATGTATCTGTTGTGG	GGTGGAACTCCTTTTTTGACTGG	30	60

Asterisks indicate that a hot start PCR was required.

### Characterisation of the tammar IFITM gene cluster and FISH

To characterise the tammar *IFITM* gene cluster, a male tammar genomic BAC library obtained from Arizona Genomics Institute (Tucson, AZ, USA) was screened using ^32^P-labelled probes as described previously [34]. Probes to tammar *IFITMA2* and *IFITM10* were labelled using the Megaprime DNA labelling kit (GE Healthcare, NSW, Aust.). DNA was extracted from the resulting positive BAC clones using the PhasePrep BAC DNA kit (Sigma-Aldrich, NSW, Aust.) and the purified DNA was shotgun sequenced at the Australian Genome Research Facility (Qld, Aust.).

Fluorescence in situ hybridisation (FISH) was performed as described previously [35]. Briefly, the purified BAC genomic clones were labelled with dUTP-digoxygenin (DIG) by nick translation at 14°C for about one hour using the Megaprime DNA Labelling kit (GE Healthcare, Aust.) and then co-precipitated with tammar Cot-1 DNA. Tammar metaphase chromosomes from testis were then incubated with the labelled probes overnight at 37°C. Bound probe was detected using a mouse anti-DIG FITC–labelled antibody (Roche, NSW, Aust.) and chromosomes were counterstained with DAPI (4, 6-diamidino- 2-phenylindole).2

### Protein alignments and phylogenetic tree construction

Amino acid sequences of mouse, human and rat IFITMs were obtained from the National Centre for Biotechnology Information [32]. Genomes of the platypus, chicken, lizard, frog, fish, lamprey, sea squirt, lancelet, sea urchin, mollusc, fly and nematode were searched for *IFITM* homologues (Additional file 1: Table S 1) using the UCSC Genome Bioinformatics [29], Ensembl [31] and NCBI websites [32]. Multisequence amino acid alignments were performed using ClustalW [36] and edited with GeneDoc [37]. A phylogenetic tree of vertebrate IFITMs for which full length (or almost full length) sequence was available was constructed using Phylip [38]. First, multi-sequence alignment of amino acids was performed using ClustalW using the PAM weight matrix. Then, in Phylip, the PAM distance matrix was calculated in Protdist. The matrix was transformed into a Neighbour joining tree and then a majority-rule consensus tree for 1000 bootstraps was drawn using Consense.

### Expression analysis by RT-PCR

Tissues were obtained from tammars in our University of Melbourne breeding colony. Samples were collected from females carrying embryos or fetuses as described previously [39,40]. Briefly, for the pre-natal stages, embryos up to and including the early somite stage were frozen whole. Mid-somitogenesis stage embryos with their adjoining vascular (trilaminar) yolk sac were separated from their avascular (bilaminar) yolk sac and the two regions were frozen separately. All later stages were divided into embryo or fetus, vascular yolk sac placenta and avascular yolk sac placenta and each region was frozen separately. Total RNA was extracted from adult tissues using Tri Reagent (Ambion Inc, Texas CA., USA) and from conceptuses using the GenElute Mammalian Total RNA Miniprep kit (Sigma Aldrich, NSW, Aust.) according to kit protocols. RNA was DNAse-treated with DNA-Free (Ambion) and 40 ng of RNA was reverse-transcribed using SuperScript III (Invitrogen, CA, USA) in a total volume of 20 μL. PCR was performed using GoTaq (Promega, NSW, Aust.) in a 30 μL reaction, which included 0.5 μL of cDNA and primers at a final concentration of 0.5 μM. PCR amplification involved an initial 2 minute denaturation step at 94°C and extension at 72°C for 30 seconds. The number of amplification cycles and the annealing temperatures for each gene are listed in Table 3. All experiments were approved by the University of Melbourne Animal Experimentation Ethics Committees and all animal handling and husbandry were in accordance with the National Health and Medical Research Council of Australia (2004) guidelines.

## Abbreviations

BAC, Bacterial artificial chromosome; BLAST, Basic local alignment search tool; BYS, Bilaminar yolk sac; CD225, Cluster of differentiation antigen; EST, Expressed sequence tag; FISH, Fluorescence in situ hybridisation; IFITM, Interferon inducible transmembrane protein; ISRE, Interferon-stimulated response element; NCBI, National Centre for Biotechnology Information; ORF, Open reading frame; PGCs, Primordial germ cells; shRNA, Short hairpin RNA; UTR, Untranslated region; WGS, Whole genomic shotgun.

## Competing interests

The authors declare that they have no competing interests.

## Authors’ contributions

DH carried out the experimental work and participated in the bioinformatic analyses and drafted the manuscript. SF participated in the bioinformatic analyses. GS and MBR collected embryo specimens and SF, GS and MBR revised the manuscript. All authors read and approved the final manuscript.

## Supplementary Material

Additional file 1 **Table S1.** Summary of IFITM orthologues and paralogues in animals from various classes. Click here for file
