# Supplementary material for: Evolution of vertebrate interferon inducible transmembrane proteins
Source: BMC Genomics. 2012 Apr 26;13:155. doi: 10.1186/1471-2164-13-155 (PMC3424830; doi:10.1186/1471-2164-13-155)
Supplement: Additional file 1 — Table S1. Summary of IFITM orthologues and paralogues in animals from various classes. [file 1471-2164-13-155-S1.pdf]

Suppl. Table 1 – Summary of *IFITM* orthologues and paralogues in animals from various classes

Sub-phylum	Class	Species	Gene name	GenBank accession	Ensemble accession	Genome build	Location	Coding region predicted from genomic sequence
Cephalochordata		*Branchiostoma floridae*		BW694636		JGI 1.0/braFlo1		
Urochordata	Ascidiacea	*Ciona intestinalis**				JGI 2.1/ci2		
Vertebrata	Agnatha	*Petromyzon marinus^*				WUGSC 3.0/petMar1	Contig 20370	Ex 1 13405-13490<br>Ex 2 13378-13638
	Chondrichthyes<br>Sc Elasmobranchii#	*Torpedo marmorata*		U16697.1				
	Sc Elasmobranchii#	*Scyliorhinus torazame*		FY415144<br>FY415470				
	Sc Elasmobranchii#	*Squalus acanthias*		EE049265				
	Osteichthyes	*Danio rerio*	IFITM1<br>IFITM5	NM_001110287<br>NM_001177312		Zv9/danRer7<br>Zv9/danRer7	Chr. 5<br>Chr. 25	
		Takifugu rubripes	 <br><br>IFITM5		ENSTRUT00000041649<br>ENSTRUT00000041634<br>ENSTRUT00000041638<br>ENSTRUT00000041650	FUGU 4.0, Ver. 64.4<br>FUGU 4.0, Ver. 64.4<br>FUGU 4.0, Ver. 64.4<br>FUGU 4.0, Ver. 64.4	Scaffold 2<br>Scaffold 2<br>Scaffold 2<br>Scaffold 2	
	Amphibia<br>Sc Anura	 *Xenopus (Silurana) tropicalis*	IFITM1<br>IFITM3<br>IFITM5-like<br>IFITM3-like<br>IFITM5-like<br>IFITM5-like<br>IFITM10-like	NM_001129931<br>NM_001015758<br>XM_002937645<br>XM_002937646<br>XM_002937633<br>XM_002937643<br>XM_002938896		JGI 4.2/xenTro3<br>JGI 4.2/xenTro3<br>JGI 4.2/xenTro3<br>JGI 4.2/xenTro3<br>JGI 4.2/xenTro3<br>JGI 4.2/xenTro3<br>JGI 4.2/xenTro3	Scaffold 306<br>Scaffold 306<br>Scaffold 306<br>Scaffold 306<br>Scaffold 306<br>Scaffold 306<br>Scaffold 419	
	Sc Urodela#	*Ambystoma tigrinum tigrinum*		CN057106<br>CN061750<br>CN063982<br>CN060940<br>CN063220<br>CN065280				
		Ambystoma mexicanum		CN041559<br>CN036724<br>CN041095<br>CN046251<br>CO789273				
	Reptilia	*Anolis carolinensis*	IFITM2-like<br>IFITM5-like<br>IFITM10-like	XM_003214858<br>XM_003214793<br>XM_003214768		Broad AnoCar2.0/anoCar2<br>Broad AnoCar2.0/anoCar2<br>Broad AnoCar2.0/anoCar2	Chr. 1<br>Chr. 1<br>Chr. 1	
	Aves	*Gallus gallus*	IFITM1-like<br>IFITM5<br>IFITM10	XM_420925<br>NM_001199498<br>XM_001234445		WUGSC 2.1/galGal3<br>WUGSC 2.1/galGal3<br>WUGSC 2.1/galGal3	Chr. 5<br>Chr. 5<br>Chr. 5	
	Mammalia<br>Sc Monotremata	 *Ornithorhynchus anatinus*		 XM_001521373^		 WUGSC 5.0.1/ornAna1	 Contig 12092	

		IFITM5-like	XM_001520686	WUGSC 5.0.1/ornAna1	Contig 16321	
Sc Marsupialia	*Monodelphis domestica*	IFITMA	gnl|ti|515911565			
		IFITMB1		Broad/monDom5	Chr. Un	Ex 1 15852005-15852163<br>Ex 2 15853226-15853394
		IFITMB2		Broad/monDom5	Chr. Un	Ex 1 15998963-15999121<br>Ex 2 16000305-16000493
		IFITM5	XM_001363778		Chr. Un	
		IFITM10-like	XM_001367690		Chr. Un	
	Macropus eugenii	IFITMA1	JQ254908		Chr. 5	
		IFITMA2	JQ254909		Chr. 5	
		IFITMB	JQ254910		Chr. 5	
		IFITM5	JQ254911		Chr. 5	
		IFITM10	JQ254912		Chr. 2	
Sc Eutheria	*Homo sapien*	IFITM1	NM_003641		Chr. 11	
		IFITM2	NM_006435		Chr. 11	
		IFITM3	NM_021034		Chr. 11	
		IFITM5	NM_001025295		Chr. 11	
		IFITM10	NM_001170820		Chr. 11	
	Mus musculus	Ifitm1	NM_001112715		Chr. 7	
		Ifitm2	NM_030694		Chr. 7	
		Ifitm3	NM_025378		Chr. 7	
		Ifitm5	NM_053088		Chr. 7	
		Ifitm6	NM_001033632		Chr. 7	
		Ifitm7	NM_028968		Chr. 16	
		Ifitm10	NM_177265		Chr. 7	
	Rattus norvegicus	Ifitm1	NM_001106314		Chr. 1	
		Ifitm2	NM_030833		Chr. 1	
		Ifitm3	NM_001136124		Chr. 1	
		Ifitm5	XM_002728832		Chr. 1	
		Ifitm6	XM_219476		Chr. 1	
		Ifitm7	XM_221637		Chr. 11	
		Ifitm10	XM_215129		Chr. 1	

* no IFITM orthologues detected in this sub-phylum

# no genomic database available – results based on EST data. *IFITM* paralogs or orthogs may be identified when a genomic database is available

^ only partial sequence is available

Sc, subclass
